# Elevated fecal peptidase D at onset of colitis in Galphai2^-/-^ mice, a mouse model of IBD

**DOI:** 10.1371/journal.pone.0174275

**Published:** 2017-03-21

**Authors:** Daniel Bergemalm, Robert Kruse, Maria Sapnara, Jonas Halfvarson, Elisabeth Hultgren Hörnquist

**Affiliations:** 1 Department of Gastroenterology, Faculty of Medicine and Health, University of Örebro, Örebro, Sweden; 2 School of Medical Sciences, Faculty of Medicine and Health, University of Örebro, Örebro, Sweden; 3 Department of Microbiology and Immunology, University of Gothenburg, Institute of Biomedicine, Gothenburg, Sweden; 4 Department of Internal Medicine and Clinical Nutrition, University of Gothenburg, Institute of Medicine, Gothenburg, Sweden; Uniwersytet Gdanski, POLAND

## Abstract

**Background:**

The identification of novel fecal biomarkers in inflammatory bowel disease (IBD) is hampered by the complexity of the human fecal proteome. On the other hand, in experimental mouse models there is probably less variation. We investigated the fecal protein content in mice to identify possible biomarkers and pathogenic mechanisms.

**Methods:**

Fecal samples were collected at onset of inflammation in Galphai2^-/-^ mice, a well-described spontaneous model of chronic colitis, and from healthy littermates. The fecal proteome was analyzed by two-dimensional electrophoresis and quantitative mass spectrometry and results were then validated in a new cohort of mice.

**Results:**

As a potential top marker of disease, peptidase D was found at a higher ratio in Galphai2^-/-^ mouse feces relative to controls (fold change 27; p = 0.019). Other proteins found to be enriched in Gαi2^-/-^ mice were mainly pancreatic proteases, and proteins from plasma and blood cells. A tendency of increased calprotectin, subunit S100-A8, was also observed (fold change 21; p = 0.058). Proteases are potential activators of inflammation in the gastrointestinal tract through their interaction with the proteinase-activated receptor 2 (PAR2). Accordingly, the level of PAR2 was found to be elevated in both the colon and the pancreas of Galphai2^-/-^ mice at different stages of disease.

**Conclusions:**

These findings identify peptidase D, an ubiquitously expressed intracellular peptidase, as a potential novel marker of colitis. The elevated levels of fecal proteases may be involved in the pathogenesis of colitis and contribute to the clinical phenotype, possibly by activation of intestinal PAR2.

## Introduction

Inflammatory bowel disease (IBD), mainly ulcerative colitis (UC) and Crohn’s disease (CD), is a potentially devastating disease affecting individuals of all ages. IBD has a peak onset in young adults, and is often characterized by a chronic course with flares and symptom-free periods [1]. The combination of young onset and an expected normal lifespan means a high degree of accumulated morbidity and appreciable costs. Establishment of the diagnosis and monitoring of the disease have traditionally been based on clinical parameters, endoscopic evaluation, imaging, and nonspecific blood markers such as C-reactive protein, hemoglobin, and albumin. In recent years, fecal markers such as calprotectin have been shown to discriminate between functional disorders and IBD, and have recently been proposed to be reliable markers for early detection of relapse and for identification of patients with an increased risk of a future flare [2, 3]. The concept of using novel fecal protein markers for, for example, diagnosis, monitoring of disease severity, and drug efficacy is promising as it would be non-invasive and easily accessible. However, the variation in food intake and gut microbiota between individuals would be expected to cause a high degree of complexity in the fecal proteome [4]. These concerns may hinder the discovery of new markers, particularly when performed in humans―even though some recent advances have been reported [5]. In contrast, laboratory mouse models have a controlled environment with minimal variation in food and exposure to bacteria, so they could be expected to have less variable fecal proteomes.

Knockout of the heterotrimeric G-protein subunit Galphai2 in mice results in a phenotype dominated by severe and chronic colitis resembling IBD [6]. The lethal inflammation in mice with a 129Sv background starts from 6‒12 weeks of age and includes bloody diarrhea, colon distension, peritonitis, mucosal dysplasia, and colorectal cancer, whereas mice with a C57Bl/6 background remain free of colitis. The inflammation is characterized by proinflammatory Th1 lymphocytes at advanced stages of the disease [7], but in pre-symptomatic mice there are other changes including elevated quantities of antibodies to commensal microbiota, dietary products, and self-tissues―including perinuclear anti-neutrophil cytoplasmic antibodies (p-ANCA) [8, 9]. Bone marrow-derived Galphai2^-/-^ cells mediate colitis, which has been shown by reciprocal bone marrow transfer between Galphai2^-/-^ and wild-type mice [10]. More recent data by us have revealed an early and dramatic increase in Th17-associated cytokine responses as well as alterations of the microbiota related to the degree of inflammation in the Galphai2^-/-^ model [11, 12].

Proteomics is the ideal method for identification of previously unknown protein markers. The effect of colitis on the fecal proteome has not yet been analyzed in a controlled system such as a laboratory mouse model. Only a few studies have analyzed the fecal proteome of laboratory mice, but to our knowledge never in the context of IBD [4, 13, 14]. Also, since all Galphai2^-/-^ mice eventually develop colitis, mice predestined for disease but still in the pre-symptomatic phase can be studied.

In the present study, we aimed to investigate any alterations in the fecal proteome caused by *Galphai2* knockout, in order to identify possible biomarkers and gain a better understanding of the pathogenic events that cause IBD-related manifestations in this model.

## Materials and methods

### Animals

Galphai2^-/-^ mice with a pure 129SvEv background were bred using heterozygous (Galphai2^+/-^ males and females. The animals were kept at the Department of Experimental Biomedicine, University of Gothenburg. Polymerase chain reaction on genomic DNA from the tail was used to verify homozygosity. Heterozygous (Galphai2^+/-^) or wild-type animals did not develop any obvious phenotype, and had a normal lifespan. Homozygous animals developed severe diarrhea starting around six weeks of age, and succumbed to colitis within a few weeks. The mice were monitored daily by experienced animal keepers who have oral and written information about this specific genotype and symptoms of disease, and were euthanized if they presented with a reduced general condition such as erected fur and/or altered social behavior, or had lost more than 15% of their body weight. They were bred and kept in filter top micro isolator cages, at all occasions accompanied by other mice. They received environmental enrichment in the form of e.g. nest pads, and were given food and water ad libitum. None of the mice in the study died without euthanasia as a result of severe colitis. The mice were not euthanized for the collection of feces. Mice presenting with symptoms of severe colitis as described above were euthanized by cervical dislocation, followed by removal of the heart. The mice were health-screened in accordance with recommendations from the Federation of European Laboratory Animal Science Associations (FELASA), and the Animal Ethics Committee of Göteborg University approved the study.

### Homogenization of tissue and fecal pellets

Proteins from fecal pellets were extracted as previously described, with some modifications [4]. Briefly, fecal pellets from male and female Galphai2^-/-^ mice were collected at suspected onset of disease and before any overt clinical signs of disease (4‒5 weeks), for proteomics. Fecal pellets from four-week-old mice were easy to handle and were homogenized individually. For validation experiments, proteins were also extracted from feces of mice with manifest colitis (6‒12 weeks old). Sampling of individual fecal pellets from a few mice with symptomatic colitis were complicated by diarrhea, but the same amount of buffer relative to fecal weight was always used for protein extraction. Pellets from wild-type and Galphai2^+/-^ littermates were collected in a similar way at the same ages. To minimize experimental variation, we only collected freshly produced pellets. This was assured by moving individual animals to new cages for collection of samples. All pellets were stored immediately at −80°C. Before homogenization, the fecal pellets were weighed and put in a 1:10 dilution (g/ml) of urea solution (8 M urea, 2 M CHAPS) for two-dimensional electrophoresis, or Tris buffer (20 mM Tris-HCl, pH 7.5, 1% Triton X-100) for quantitative mass spectrometry and immunoblotting. All samples were treated with a protease inhibitor cocktail (Sigma Aldrich), as high levels of proteases have been associated with colitis [15, 16]. The pellets were disrupted manually using the tip of a plastic pipette prior to homogenization using an Ultraturrax (IKA, Stufen, Germany).

Protein extracts from colon and pancreas for immunoblotting were prepared in a similar way in Tris buffer, using an Ultraturrax. Homogenates were then centrifuged at 20 000 *g* to pellet undissolved material and collected supernatants were kept at −80°C before analysis. The total protein content of fecal protein samples was determined using the DC protein assay (Bio-Rad, Hercules, CA, USA) or 2D Quant Kit (GE Healthcare, Uppsala, Sweden). Protein concentrations were generally about 2‒2.5 mg/ml, with a trend towards a somewhat lower concentration in Galphai2^-/-^ mice.

### Two-dimensional gel electrophoresis (2DE)

To get an overview of protein distribution, individual sample variation and semi-quantitative measures of individual protein spots, two-dimensional gel electrophoresis was performed as previously described [17]. Thirty μg of individual fecal protein samples from four-week-old Galphai2^-/-^ animals, with no or only early signs of colitis, as well as similar protein samples from age-matched healthy animals of Galphai2^+/-^ and wild-type background, were supplied with pH 3-11NL IPG buffer (GE Healthcare) to a final concentration of 0.5% and rehydration buffer (8 M urea, 2% CHAPS, and 0.002% bromophenol blue) to a final volume of 200 μl. The samples were loaded and rehydrated into 11-cm 3-11NL IPG strips (GE Healthcare) for 12–16 h. Isoelectric focusing (IEF) was done using a Protean IEF cell (Bio-Rad) at 20°C, and the maximum current was set to 50 μA per strip with increase of voltage stepwise until 11 000 Volt-hours was reached. Following IEF, the strips were equilibrated for 15 min in equilibration buffer (6 M urea, 50 mM Tris-HCl, pH 8.8, 30% (v/v) glycerol, 2% SDS, and 0.002% bromophenol blue) containing 1% dithiothreitol, and then for 15 min with 2.5% iodoacetamide in the same buffer. Second-dimension electrophoresis was run in a Criterion^TM^ Cell (Bio-Rad) electrophoresis system. The IPG strips were placed on top of 10‒20% (w/v) gradient acrylamide gels (Bio-Rad) and sealed with 1% melted agarose in SDS running buffer. Gels were run at 20°C and 200 V for one hour. For analysis, they were stained with Oriole™ fluorescent dye (Bio-Rad) and excited by UV-light using a Chemidoc XRS imager (Bio-Rad) with emission filter at 580 nm.

### Quantitative mass spectrometry (QMS) using Tandem Mass Tags (TMT)

#### Sample preparation for mass spectrometric analysis

Proteins were extracted from feces from three 4-week-old Galphai2^-/-^ animals with early signs of colitis and from wild-type littermates, matched by age, by homogenization in extraction buffer (20 mM Tris-HCl and 1% Triton X-100) containing protease inhibitor cocktail (Sigma Aldrich). The total protein content was determined with the Pierce™ BCA Protein Assay (Thermo Scientific, Waltham, MA, USA). Samples (45 μg) were reduced by addition of 2M DL-dithiothreitol to a final concentration of 100 mM and incubated at 60°C for 30 min. The samples were trypsin digested using the filter-aided sample preparation (FASP) method modified from Wisniewski et al [18]. Briefly, reduced samples were applied to Nanosep 10k Omega filters (Pall Life Sciences, and repeatedly washed with 8 M urea. Alkylation was performed with methyl methane thiosulfonate (MMTS) diluted in digestion buffer (1% sodium deoxycholate and 20 mM TEAB) for 20 min at RT, which was followed by washing with digestion buffer. Trypsin (Pierce Trypsin Protease, MS grade; Thermo Scientific, Waltham, MA, USA) at a ratio of 1:100 relative to the protein content was added in 50 mM TEAB to a pH of about 8, and the samples were incubated overnight at 37°C. Another portion of trypsin was added and the mixture was incubated at 37°C for three hours. The peptides were collected by centrifugation and subjected to isobaric mass tagging reagent, TMT®, according to the manufacturer’s instructions (Thermo Scientific). After TMT labeling, the samples were pooled and acidified with formic acid to precipitate sodium deoxycholate.

The peptides were further purified and fractionated by strong cation exchange chromatography (ÄKTA-system; Amersham-Pharmacia) on a PolySULFOETHYL A™ column (100 × 2.1mm, 5 μm, 300 Å; PolyLC Inc.). Solvent A was 25 mM ammonium formate, pH 2.8, and solvent B was 500 mM ammonium formate, pH 2.8. The gradient was run at 0.25 ml/min; 20% solvent B over 20 min, 40% solvent B over 10 min, and finally 100% solvent B over 10 min. In total, 28 fractions were collected and 22 fractionations containing the peptides from each set were desalted using PepClean C18 spin columns (Thermo Fisher Scientific) according to the manufacturer’s guidelines.

#### LC-MS/MS analysis

The dried, desalted 10-plexed TMT-labeled sample was reconstituted with 15 μl of 0.1% formic acid (Sigma Aldrich) in 3% acetonitrile and analyzed on an Orbitrap Fusion Tribrid mass spectrometer interfaced to an Easy-nLC II (Thermo Fisher Scientific). Peptides (2 μl injection volume) were separated using an in-house constructed analytical column (300 × 0.075 mm internal diameter) packed with 1.8 μm Reprosil-Pur C18-AQ particles (Dr. Maisch, Germany). Solvent A was 0.2% formic acid in water and solvent B was 0.2% formic acid in acetonitrile. The following gradient was run at 200 nl/min: 5‒30% solvent B over 75 min and 30‒80% solvent B over 5 min, with a final hold at 80% solvent B for 10 min. Ions were injected into the mass spectrometer under a spray voltage of 1.6 kV in positive ion mode. MS scans were performed at 120 000 resolution, m/z range 350‒1,500. MS/MS analysis was performed in a data-dependent multinotch mode, with a top speed cycle of 3 s for the most intense doubly or multiple charged precursor ions. Ions in each MS scan over threshold 5,000 were selected for fragmentation (MS2) by collision-induced dissociation (CID) for identification at 30% and detection in the ion trap followed by multinotch (simultaneous) isolation of the top 10 MS2 fragment ions, with m/z 400‒900, selected for fragmentation (MS3) by high energy collision dissociation (HCD) at 55% and detection in the Orbitrap at 60 000 resolution, m/z range 100‒500. Precursors were isolated in the quadrupole with a 1.6 m/z window and dynamic exclusion within 10 ppm during 30 s was used for m/z-values already selected for fragmentation.

#### Data processing and quantitative analysis

MS raw data files for each TMT set were merged for relative quantification and identification using Proteome Discoverer version 1.4 (Thermo Fisher Scientific). A database search for each set was performed with the Mascot search engine (Matrix Science) using the *Mus musculus* Swissprot Database version November 2014 (Swiss Institute of Bioinformatics, Switzerland) with MS peptide tolerance of 10 ppm and MS/MS tolerance for identification of 500 millimass units (mmu). Tryptic peptides were accepted with zero missed cleavage and variable modifications of methionine oxidation and cysteine alkylation, and fixed modifications of N-terminal TMT-label and lysine TMT-label were selected. The detected peptide threshold in the software was set to 1% False Discovery Rate by searching against a reversed database, and proteins identified were grouped by sharing the same sequences to minimize redundancy. For TMT quantification, the ratios of the TMT reporter ion intensities in HCD MS/MS spectra (m/z 126‒131) from raw data sets were used. Ratios were derived by Proteome Discoverer using the following criteria: fragment ion tolerance as 3 mmu for the most confident centroid peak and missing values are replaced with minimum intensity. Only peptides unique to a given protein were considered for relative quantitation, excluding those common to other isoforms or proteins of the same family. The quantification was normalized using the applied wet-weight of feces and the total concentration of the protein. Missing proteins from individual animals were substituted with a background value estimated from noise. For quantitative analysis, the MS3 spectra were evaluated manually.

### Immunoblotting

Sodium dodecyl sulfate-polyacrylamide gel electrophoresis (SDS-PAGE) was performed according to standard procedures. Briefly, the same amount of μg of individual protein samples were supplied with Laemmli buffer (2% SDS, 5% 10-mercaptoethanol, 10% glycerol, 0.002% bromophenol blue, 62.5 mM Tris-HCl pH 6.8) and loaded in each well for separation on pre-made Criterion 10‒20% gradient polyacrylamide gels (Bio-Rad), and further electroblotted onto polyvinylidine difluoride membranes (GE Healthcare). Blots were probed with monoclonal primary anti-trypsin (1:1 000; Abcam, Cambridge, UK), monoclonal primary anti-PAR2 (1:2 000; Abcam), or polyclonal anti-PEPD (1:500; Sigma Aldrich) antibody followed by horseradish peroxidase-coupled secondary anti-mouse IgG or anti-rabbit IgG antibody (1:100,000 dilution; GE Healthcare) and visualized using chemiluminescence using ECL prime (GE Healthcare). Bands were captured using a Chemidoc XRS imager (Bio-Rad) and densiomteric quantitation were performed using Image Lab software v. 5.0 (Bio-Rad).

### Statistical analysis and annotation of protein function and subcellular location

Student’s t-test was used for statistical analyses of quantitative mass spectrometry. Statistical significance were set at a p < 0.05 and biological significance was set at a fold change of ≥ 2 between groups, or ≥ 2 as compared to background noise.

Individual densiometric data from the immunoblots, were divided by the mean of protein levels of wild-type mice or pre-symptomatic mice as specified in each figure. These relative protein values were imported into SPSS version 22 (IBM) for statistical comparison using the Mann-Whitney non-parametric test.

For functional and subcellular compartment analysis, the software Ingenuity Pathway Analysis knowledge base (December 5, 2015) [19] was used together with information from the UniProt knowledge database [20] and the Human Protein Atlas [21].

## Results

### Isolation of fecal protein

All Galphai2^-/-^ mice were born healthy and developed obvious signs of colitis (diarrhea, blood in stool) at 5‒6 weeks of age, as previously described [22]. To identify protein markers reflecting onset of disease, fecal samples were obtained at four to five weeks of age. At this age, the mice did not show any signs of colitis but had slightly softer fecal pellets. It can be speculated that this point in time correspond to when patients with suspected IBD are referred to specialist care and could therefore be of clinical relevance for identification of possible diagnostic markers.

### Proteomic analysis

#### Two-dimensional electrophoresis

**Fig 1:**
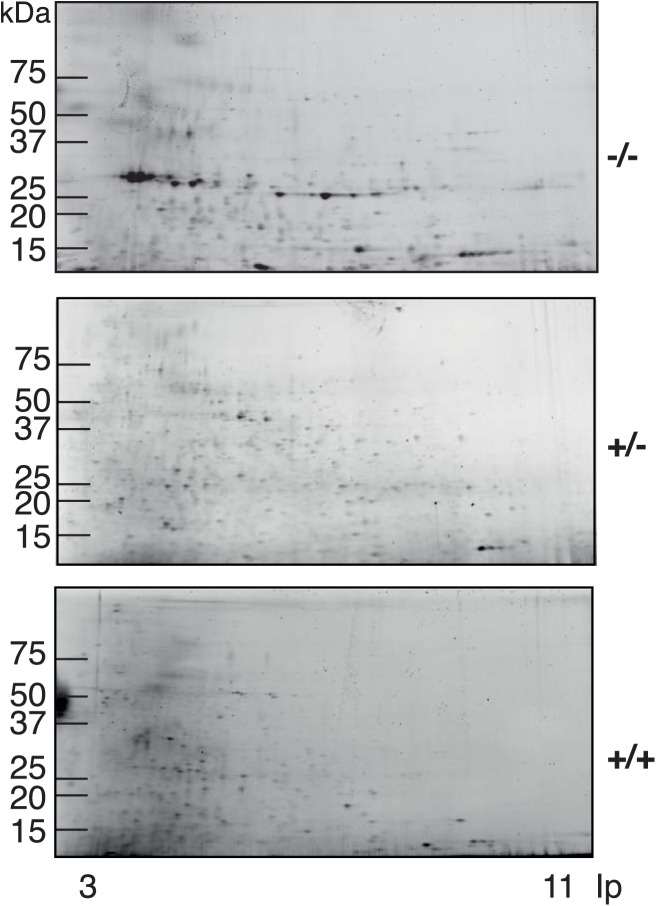
Two-dimensional electrophoresis of fecal protein samples of Galphai2^-/-^ mice, Galphai2^+/-^ mice, and wild-type littermates. Samples were run on gels with broad coverage of isoelectric points (3‒11) and molecular weights. A few spots of high intensity, that markedly differed from their heterozygous and wild-type littermates, predominated in the gels from Galphai2^-/-^ mice.

Feces from Galphai2^-/-^ mice, healthy Galphai2^+/-^ mice, and wild-type littermates were homogenized and the proteins separated by 2DE (Fig 1).

Gels showed a reproducible spot pattern although slightly weaker than expected from the applied amount of total protein. In average about 400 individual protein spots were recognized from fecal samples of healthy Galphai2^+/-^ mice and wild-type littermates whereas Galphai2^-/-^ mice showed somewhat fewer spots in the 2DE separation.

#### Quantitative mass spectrometry

For identification and quantification of potential biomarkers, we then analyzed another set of fecal samples from four-week-old Galphai2^-/-^ and wild-type mice by QMS. We choose to focus on host proteins only, so the data search was done against a mouse proteome database for protein identification. In total, 120 murine proteins were identified; ten of these proteins were unique to fecal samples from Galphai2^-/-^ mice whereas only one protein was unique to the healthy littermates (S1 Table). However, the difference in individual protein levels between the two groups was greater than a fold change of +/- 2 and statistically significant for ten proteins only, and seven other proteins were found to be borderline significant (Table 1). Of interest, the concentration of peptidase D (Xaa-Pro dipeptidase) was high in Galphai2^-/-^ mice feces but could not be detected in any of the wild-type mice, corresponding to a 27-fold increase in Galphai2^-/-^ mice relative to the observed background noise in healthy littermates (p = 0.019). A possible increase in protein S100-A8 (fold change = 21, p = 0.058), known to be one of the subunits of calprotectin, could be seen in Galphai2^-/-^ mice compared to wild-type mice (Table 1).

**Table 1:** Top fecal proteins that were identified to be upregulated or downregulated in feces of Galphai2^-/-^ mice.

UniProtKB ID	Protein name	FC	p	Function	Origin
P97449	Aminopeptidase N	4.7	0.00004	Protease	GI/Blood cells
Q9R100	Cadherin-17	4.1	0.0001	Cell adhesion	GI tract
P07309	Transthyretin	11.2	0.003	Transport	Plasma
P28843	Dipeptidyl peptidase 4	2.1	0.006	Protease	Small intestine
P60710	Actin, cytoplasmic 1	−2.3	0.008	Cytoskeleton	GI tract
Q11136	Peptidase D	27.1	0.019	Protease	GI tract
Q6Q473	Calcium-activated chloride channel regulator 4	2.3	0.026	Protease	GI tract
P24822	Intestinal-type alkaline phosphatase	3.1	0.028	Enzyme	Small intestine
P97429	Annexin A4	2.2	0.038	Exocytosis	GI tract
P18761	Carbonic anhydrase 6	−2.5	0.049	Carbon dehydratase	Salivary glands
P17047	Lysosome-associated membrane glycoprotein 2	−3.3	0.054	Protein transport	GI/blood cells
P27005	Protein S100-A8 (calprotectin)	21	0.058	Inflammation	Neutrophils
Q9DA19	Corepressor interacting with RBPJ 1	3.0	0.079	Transcription	Pancreas
Q91XA9	Acidic mammalian chitinase	4.0	0.082	Chitinase	GI tract
Q921I1	Serotransferrin	4.3	0.087	Transport	Plasma
Q60928	Gamma-glutamyltranspeptidase 1	3.3	0.095	Protease	Pancreas/stomach
Q9JJF3	Bifunctional lysine-specific demethylase	−2.2	0.095	Demethylase	GI tract

FC = fold change.

### Data analysis

#### Subcellular origin

**Fig 2:**
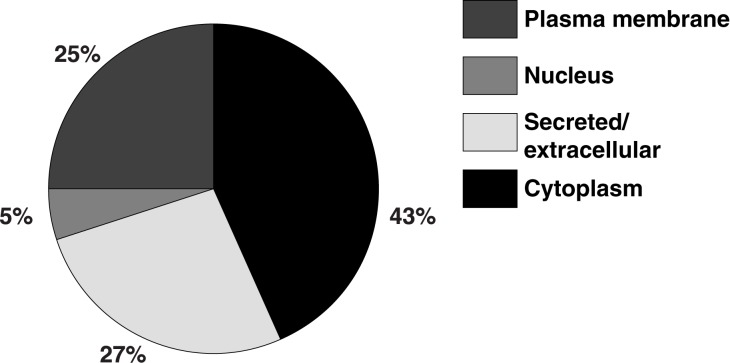
Subcellular distribution of proteins identified in feces from Galphai2^-/-^ mice and healthy mice.

All fecal proteins (n = 120) identified from QMS were analyzed further with IPA. The results show that the proteins originated from different subcellular compartments (Fig 2). Most proteins were classified as being intracellular (cytosolic or nuclear), 48%, and to a lesser extent as being derived from the plasma membrane (25%) or secreted (27%).

#### Functional groups of proteins

To classify the fecal proteins that were found to be significantly (p<0.05) or possibly (p = 0.05–0.1) altered (n = 17) in the Galphai2^-/-^ mice according to function and tissue of origin, the Ingenuity Pathway Analysis [19], the UniProt database [20], and the Human Protein Atlas were used [21] (Table 1). Seven out of the 13 proteins with higher concentrations in the feces of the Galphai2^-/-^ mice were categorized as digestive, with most of them (n = 5) being proteases (Table 1). Among the proteases, the most prominent finding was the significant elevation in peptidase D levels. Two proteins, transthyretin (p = 0.003) and serotransferrin (p = 0.087) were from plasma. The other proteins had diverse functions and could not easily be classified in common groups (Table 1).

### Validation

#### Peptidase D as a marker of colitis

**Fig 3:**
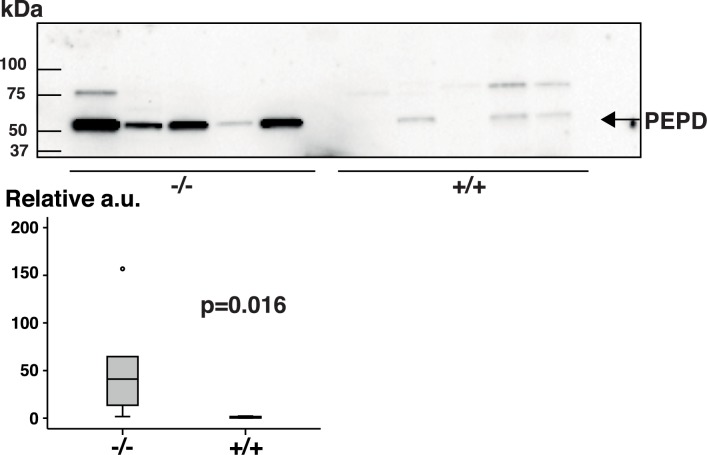
Immunoblots of peptidase D. A new set of fecal protein samples from Galphai2^-/-^ mice and healthy littermates were subjected to immunoblotting for detection of peptidase D. Equal amounts of fecal protein were loaded from five Galphai2^-/-^ mice at different stages of colitis. The first two lanes (from left) were from 4-week-old pre-symptomatic mice whereas lanes 3‒5 were from 7-, 10-, and 7-week-old mice with manifest colitis. Healthy mice were all 4 weeks old at the time of sample collection. The boxplot show densitometric quantitation of blots where peptidase D levels of Galphai2^-/-^ mice were depicted relative to the mean value of the healthy wild-type littermates. a.u. = arbitrary units.

To validate peptidase D as a potential marker of colitis, feces from new cohorts of Galphai2^-/-^ mice and wild-type littermates were collected and protein extracts were subjected to immunoblotting using an anti-peptidase D antibody (Fig 3).

The results were in accordance with the findings from QMS, but in one of the Galphai2^-/-^ mice the signal was weaker―with intensity similar to that in feces from wild-type mice. However, this mouse was older than the other mice and suffered from severe colitis, which may have influenced the results. In contrast to QMS a weak band was also detected in samples from wild-type mice, indicating that a low level of peptidase D protein was part of the normal fecal proteome.

#### Fecal proteases

**Fig 4:**
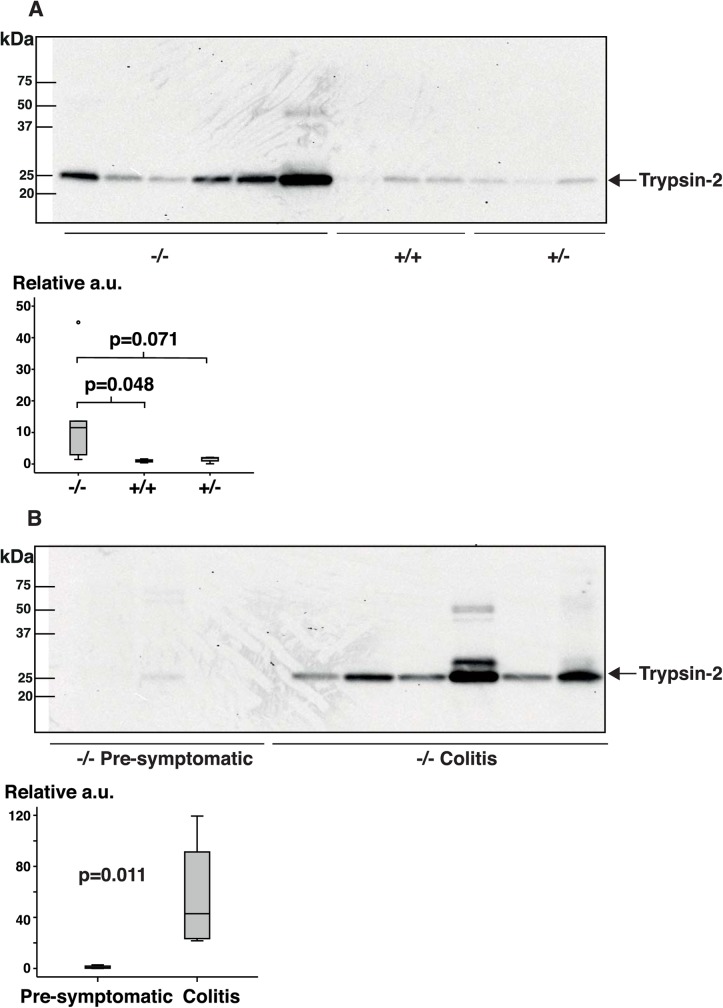
Immunoblots of trypsin-2. Fecal protein samples from Galphai2^-/-^ mice, Galphai2^+/-^ mice, and wild-type littermates were subjected to immunoblotting for detection of trypsin-2. (A) Equal amounts of fecal protein were loaded from Galphai2^-/-^ mice with early signs of colitis, along with samples from Galphai2^+/-^ mice and wild-type littermates of similar ages. (B) Fecal protein samples from Galphai2^-/-^ mice at different stages of colitis showed a tendency towards higher trypsin levels with advanced disease. The boxplots show densitometric quantitation of blots where trypsin-2 levels of Galphai2^-/-^ mice were depicted relative to the mean value of the healthy wild-type littermates (A) or the pre-symptomatic Galphai2^-/-^ mice (B). a.u. = arbitrary units.

The largest functional group of altered proteins in Galphai2^-/-^ feces was proteases. Increased amounts of the serine protease pancreatic trypsin have consistently been associated with IBD [15, 16]. Although the level/abundance of pancreatic trypsin was not significantly increased in our QMS analyses, we estimated the amount of trypsin-2 in feces from Galphai2^-/-^ mice, Galphai2^+/-^ mice, and wild-type littermates by immunoblot (Fig 4).

**Fig 5:**
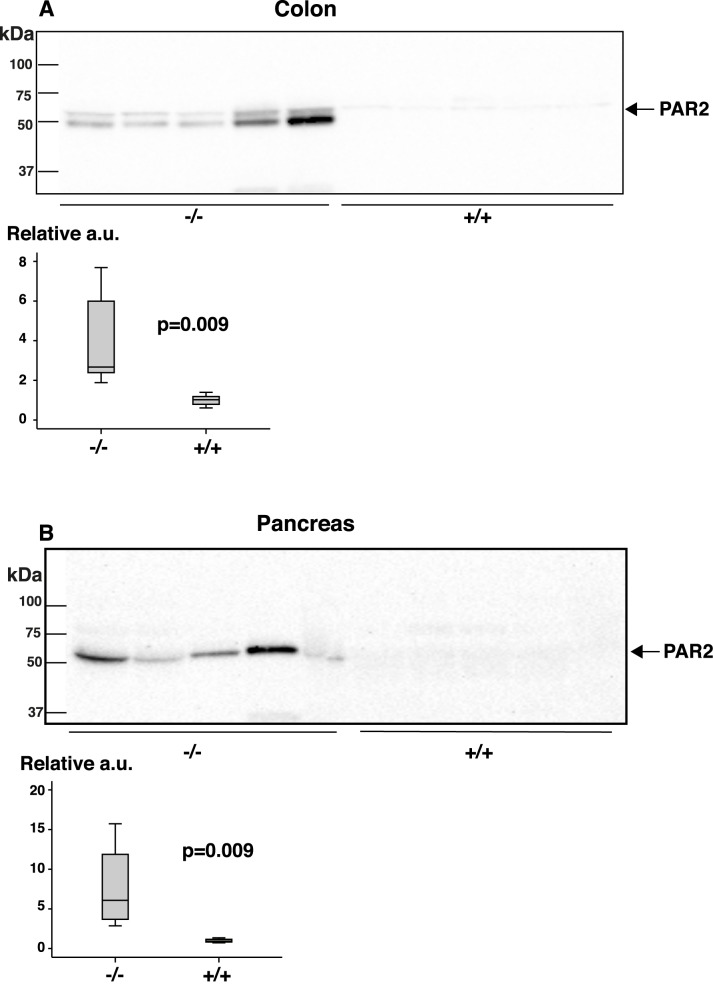
PAR2 immunoblots of homogenates from colonic and pancreatic tissue of Galphai2^-/-^ mice and healthy littermates. To examine the possible effect of increased amounts of luminal proteases on PAR2 levels, all Galphai2^-/-^ mice samples loaded on gels were from mice of different stages of clinically observable colitis (> 5 weeks). **A.** PAR2 in Galphai2^-/-^ and wild-type mice colonic homogenates. The band of slightly lower molecular weight was a cross-reacting protein of the anti-mouse secondary antibody and was excluded from the densitometric quantitation. **B.** PAR2 in Galphai2^-/-^ and wild-type mice pancreatic homogenates. Relative to Galphai2^-/-^ the PAR2 signal was weak in wild-type mice. The boxplots show densitometric quantitation of blots where PAR2 levels of Galphai2^-/-^ mice were depicted relative to the mean value of the healthy wild-type littermates. a.u. = arbitrary units.

In general, the levels of trypsin-2 were higher in feces from symptomatic Galphai2^-/-^ mice than in feces from wild-type and pre-symptomatic mice. Serine protease and other proteases are known to interact and activate proteinase-activated receptor-2 (PAR2) in the gastrointestinal tract [23, 24]. This has been suggested to contribute to the pathogenesis of IBD [23, 25, 26]. To investigate this mechanism in Galphai2^-/-^ mice, we assessed the quantity and distribution of PAR2 in the colon of mice at different stages of disease using an anti-PAR2 antibody (Fig 5A).

In colon, the PAR2 levels were similar between individual wild-type animals whereas the variation was greater in individual Galphai2^-/-^ mice. On average, Galphai2^-/-^ mice had PAR2 levels that were about three-four times higher. PAR2 is also known to be expressed in pancreatic acinar cells, where activation of the receptor is involved in the release of pancreatic enzymes [26, 27]. We therefore subjected pancreatic homogenates from symptomatic Galphai2^-/-^ mice to immunoblotting using an anti-PAR2 antibody (Fig 5B). In Galphai2^-/-^ mice, the PAR2 levels were clearly upregulated (with an average fold change of about 8), but with variation between individual mice.

## Discussion

To our knowledge, this study is the first proteomic analysis of feces in a mouse model of IBD. We have identified a potential new fecal biomarker, peptidase D, which was found in feces from Galphai2^-/-^ mice at even higher relative quantities than the S100-A8 protein, which is part of the calprotectin complex. In accordance with previous studies, we also observed elevated levels of fecal proteases and upregulation of tissue PAR2. Activation of PAR2 may result in inflammatory responses as part of the pathogenesis of IBD.

Our 2DE data indicated that the fecal proteome of Galphai2^-/-^ mice contained less protein spots than wild-type mice. This could be due to elevated levels of luminal proteases, and thereby increased degradation of proteins. However, the variations in total protein concentration of the fecal homogenates were quite small (2‒3 mg/ml, which is similar to what would be expected from tissue preparations), regardless of the method of choice for quantification. From both 2DE and QMS analyses, the individual protein levels and estimated amounts of total protein were repeatedly lower than expected from what was theoretically applied by measurements of the total protein concentration of the fecal extracts. The reason for this discrepancy is unclear, but it could partly be due to high amounts of small, partially degraded peptides outside the range of the proteomic analysis. We could also expect that a significant proportion of the fecal proteins were derived from microbiota or digested food and therefore not identified from search against a mouse database.

The murine fecal proteome has previously been examined for identification of possible biomarkers of colorectal adenomas [13]. Ang et al. identified 115 proteins of murine origin, which was similar to the number of proteins identified in the present study. Approximately one-third of the proteins identified from their model overlapped with the findings in our study. Lichtman et al. mapped the diversity of the fecal proteome in response to different microbiota, and reported a core fecal proteome of 68 proteins [4]. Twenty-eight out of these 68 “core” proteins were identified in our dataset, but none of the proteins unique to Galphai2^-/-^ mice were regarded as part of the core proteome. In our study, proteins predicted to be secreted or extracellular made up only 27% of the proteins identified, which is lower than what would be expected from earlier studies [13]. The apparent increase in luminal proteins of intracellular origin (Fig 2) may be due to mucosal damage in Galphai2^-/-^ mice suffering from inflammation, despite having a minimum of clinical signs of disease, but could also derive from infiltrative white blood cells. The modest overlap between the Galphai2^-/-^ model and previous studies on murine models of colorectal cancer is probably related to mouse model-specific differences. However, the influence of possible animal facility-specific differences might also affect the variation.

Peptidase D is an intracellular peptidase, primarily involved in the degradation of collagen but to some extent also of nutritional proteins―for salvage of the amino acid proline. Germ-line mutations causing loss of protein activity are known to result in the recessively inherited disorder prolidase deficiency [28]. The protein gets activated through phosphorylation and is regulated mainly through β1 integrin and collagen-1 signaling [29, 30]. How peptidase D ends up in the intestinal lumen and feces is unclear; it could of course be by active transport or as debris from necrotic mucosal cells. The potential of peptidase D as a fecal marker of colitis needs to be confirmed by further studies in humans, which was beyond the scope of this study. Peptidase D, however, appears to be a promising marker since we found that the protein had an even higher signal-to-noise ratio than protein S100-A8 at the onset of disease.

The question of what triggers inflammation in the Galphai2^-/-^ mouse model still need to be answered. An increase in luminal proteases of, for example, pancreatic and microbial origin does not appear to be specific to this model, as this has already been reported in human IBD [15, 16]. Here we found evidence of apparent upregulation or decreased elimination of proteases prior to obvious clinical signs of disease, such as pronounced diarrhea. Proteases including trypsin and tryptase are known to activate PAR2, which is distributed along the gastrointestinal tract [24, 26]. In mice, administration of PAR2 agonists in the colon has been shown to upregulate PAR2 expression and induces inflammation [25]. In addition, in a PAR2 knockout model, chemically induced colitis (e.g. using dextran sodium sulfate) results in a significantly reduced phenotype [31]. A PAR2 antagonist has also been shown to be an effective treatment for colitis in rat models of IBD [32]. How these findings correlate to the pathogenic events of human IBD requires further investigation, but an increase in expression of intestinal PAR2 has been shown in tissue from both CD and UC patients [33, 34]. Regulation of this pathway is apparently a common feature of rodent IBD models of widely different background, and here we found it to be regulated in colitis caused by the *Galphai2* deletion.

In conclusion, we have identified a possible new fecal marker of colitis; peptidase D. Future studies of its potential as a biomarker of IBD in humans are essential. We have also identified a possible pathogenic mechanism of *Galphai2* knock-out, i.e. increase in fecal proteases and upregulation of PAR2 levels. The importance of PAR2 has previously been reported in other murine models as well as in patients with IBD. However, if these alterations are caused by the knockout of *Galphai2* per se or by other secondary effects requires further investigation.

## Supporting information

S1 Table The 120 identified proteins from feces of Galphai2^-/-^ mice and healthy littermates. (DOCX)
