# Supplementary material for: Elevated fecal peptidase D at onset of colitis in Galphai2-/- mice, a mouse model of IBD
Source: PLoS One. 2017 Mar 21;12(3):e0174275. doi: 10.1371/journal.pone.0174275 (PMC5360340; doi:10.1371/journal.pone.0174275)
Supplement: S1 Table — (DOCX) [file pone.0174275.s001.docx]

| S1 Table | |  |  |  |  |
| --- | --- | --- | --- | --- | --- |
| Present in both Gai2-/- and wild-type | | **Present only in wild-type** | | **Present only in Gai2-/-** | |
| Accession  (UniProtKB) | **Description** | **Accession**  **(UniProtKB)** | **Description** | **Accession**  **(UniProtKB)** | **Description** |
| O70456 | 14-3-3 protein sigma | Q9QXN0 | Protein Shroom3 | Q07076 | Annexin A7 |
| Q91XA9 | Acidic mammalian chitinase] |  |  | Q8VHF2 | Cadherin-related family member 5 |
| P68134 | Actin, alpha skeletal muscle |  |  | P62631 | Elongation factor 1-alpha 2 |
| P60710 | Actin, cytoplasmic 1 |  |  | E9PV24 | Fibrinogen alpha chain |
| P03958 | Adenosine deaminase] |  |  | P16858 | Glyceraldehyde-3-phosphate dehydrogenase |
| Q00896 | Alpha-1-antitrypsin 1-3 |  |  | P04104 | Keratin, type II cytoskeletal 1 |
| Q61838 | Alpha-2-macroglobulin |  |  | P19467 | Mucin-13 |
| P17182 | Alpha-enolase |  |  | Q922R8 | Protein disulfide-isomerase A6 |
| P97449 | Aminopeptidase N] |  |  | Q9JLT2 | Trehalase |
| Q8R0I0 | Angiotensin-converting enzyme 2 |  |  | Q11136 | Xaa-Pro dipeptidase |
| P09470 | Angiotensin-converting enzyme] |  |  |  |  |
| P07146 | Anionic trypsin-2 |  |  |  |  |
| P97429 | Annexin A4 |  |  |  |  |
| P84091 | AP-2 complex subunit |  |  |  |  |
| Q9Z2W0 | Aspartyl aminopeptidase |  |  |  |  |
| Q9CR84 | ATP synthase F(0) complex subunit C1, mitochondrial |  |  |  |  |
| Q6A051 | Attractin-like protein 1 |  |  |  |  |
| Q9JJF3 | Bifunctional lysine-specific demethylase and histidyl-hydroxylase NO66 | | |  |  |
| P09803 | Cadherin-1 |  |  |  |  |
| Q9R100 | Cadherin-17 |  |  |  |  |
| Q9D7Z6 | Calcium-activated chloride channel regulator 1 |  |  |  |  |
| Q6Q473 | Calcium-activated chloride channel regulator 4 |  |  |  |  |
| P62204 | Calmodulin |  |  |  |  |
| P14211 | Calreticulin |  |  |  |  |
| P13634 | Carbonic anhydrase 1 |  |  |  |  |
| P18761 | Carbonic anhydrase 6 |  |  |  |  |
| P23953 | Carboxylesterase 1C |  |  |  |  |
| P31809 | Carcinoembryonic antigen-related cell adhesion molecule 1 | |  |  |  |
| O35744 | Chitinase-3-like protein 3 |  |  |  |  |
| Q91VA1 | Choline transporter-like protein 4 |  |  |  |  |
| Q9CZU6 | Citrate synthase, mitochondrial |  |  |  |  |
| Q9CQC2 | Colipase |  |  |  |  |
| P01027 | Complement C3 |  |  |  |  |
| Q9DA19 | Corepressor interacting with RBPJ 1 |  |  |  |  |
| P07310 | Creatine kinase M-type |  |  |  |  |
| Q9CPY7 | Cytosol aminopeptidase |  |  |  |  |
| Q60997 | Deleted in malignant brain tumors 1 protein |  |  |  |  |
| P28843 | Dipeptidyl peptidase 4 |  |  |  |  |
| Q6PFD5 | Disks large-associated protein 3 |  |  |  |  |
| Q6DYE8 | Ectonucleotide pyrophosphatase/phosphodiesterase family member 3 | | |  |  |
| P58252 | Elongation factor 2 |  |  |  |  |
| Q8K0E8 | Fibrinogen beta chain |  |  |  |  |
| P11276 | Fibronectin |  |  |  |  |
| Q60928 | Gamma-glutamyltranspeptidase 1 |  |  |  |  |
| P16406 | Glutamyl aminopeptidase |  |  |  |  |
| Q61646 | Haptoglobin |  |  |  |  |
| P02088 | Hemoglobin subunit beta-1 |  |  |  |  |
| Q91X72 | Hemopexin |  |  |  |  |
| P10853 | Histone H2B type 1-F/J/L |  |  |  |  |
| P01878 | Ig alpha chain C region |  |  |  |  |
| P01749 | Ig heavy chain V region 3 |  |  |  |  |
| P06327 | Ig heavy chain V region VH558 A1/A4 |  |  |  |  |
| P01837 | Ig kappa chain C region |  |  |  |  |
| P01631 | Ig kappa chain V-II region 26-10 |  |  |  |  |
| P24822 | Intestinal-type alkaline phosphatase |  |  |  |  |
| P02535 | Keratin, type I cytoskeletal 10 |  |  |  |  |
| P19001 | Keratin, type I cytoskeletal 19 |  |  |  |  |
| Q3TTY5 | Keratin, type II cytoskeletal 2 epidermal |  |  |  |  |
| Q3UV17 | Keratin, type II cytoskeletal 2 oral |  |  |  |  |
| P11679 | Keratin, type II cytoskeletal 8 |  |  |  |  |
| P06151 | L-lactate dehydrogenase A chain |  |  |  |  |
| P17047 | Lysosome-associated membrane glycoprotein 2 |  |  |  |  |
| P14152 | Malate dehydrogenase, cytoplasmic |  |  |  |  |
| P08249 | Malate dehydrogenase, mitochondrial |  |  |  |  |
| P15089 | Mast cell carboxypeptidase A |  |  |  |  |
| Q61847 | Meprin A subunit beta |  |  |  |  |
| Q2VPU4 | MLX-interacting protein |  |  |  |  |
| Q80Z19 | Mucin-2 (Fragments) |  |  |  |  |
| P97500 | Myelin transcription factor 1-like protein |  |  |  |  |
| P05977 | Myosin light chain 1/3, skeletal muscle isoform |  |  |  |  |
| P97457 | Myosin regulatory light chain 2, skeletal muscle isoform |  |  |  |  |
| Q5SX39 | Myosin-4 |  |  |  |  |
| P13542 | Myosin-8 |  |  |  |  |
| Q7M758 | N-acetylated-alpha-linked acidic dipeptidase-like protein | |  |  |  |
| Q61391 | Neprilysin |  |  |  |  |
| Q9JHE3 | Neutral ceramidase |  |  |  |  |
| P11672 | Neutrophil gelatinase-associated lipocalin |  |  |  |  |
| P00688 | Pancreatic alpha-amylase |  |  |  |  |
| P17892 | Pancreatic lipase-related protein 2 |  |  |  |  |
| Q6P8U6 | Pancreatic triacylglycerol lipase |  |  |  |  |
| P32848 | Parvalbumin alpha |  |  |  |  |
| Q9JJ00 | Phospholipid scramblase 1 |  |  |  |  |
| O70570 | Polymeric immunoglobulin receptor |  |  |  |  |
| P09103 | Protein disulfide-isomerase |  |  |  |  |
| P14069 | Protein S100-A6 |  |  |  |  |
| P27005 | Protein S100-A8 |  |  |  |  |
| Q14BI7 | Putative ATP-dependent RNA helicase TDRD9 |  |  |  |  |
| P52480 | Pyruvate kinase PKM |  |  |  |  |
| Q8CJ96 | Ras association domain-containing protein 8 |  |  |  |  |
| Q9JLC8 | Sacsin |  |  |  |  |
| Q8R429 | Sarcoplasmic/endoplasmic reticulum calcium ATPase 1 | |  |  |  |
| Q921I1 | Serotransferrin |  |  |  |  |
| P07724 | Serum albumin |  |  |  |  |
| P14094 | Sodium/potassium-transporting ATPase subunit beta-1 | |  |  |  |
| Q9JIP7 | Solute carrier family 15 member 1 |  |  |  |  |
| Q80VP2 | Spermatogenesis-associated protein 7 homolog |  |  |  |  |
| P08228 | Superoxide dismutase [Cu-Zn] |  |  |  |  |
| Q9DBX3 | Sushi domain-containing protein 2 |  |  |  |  |
| Q8R3G9 | Tetraspanin-8 |  |  |  |  |
| Q93092 | Transaldolase |  |  |  |  |
| P40142 | Transketolase |  |  |  |  |
| P07309 | Transthyretin |  |  |  |  |
| P17751 | Triosephosphate isomerase |  |  |  |  |
| P58771 | Tropomyosin alpha-1 chain |  |  |  |  |
| P13412 | Troponin I, fast skeletal muscle |  |  |  |  |
| Q9QZ47 | Troponin T, fast skeletal muscle |  |  |  |  |
| Q9WTP9 | Ventral anterior homeobox 2 |  |  |  |  |
| Q62468 | Villin-1 |  |  |  |  |
| Q60932 | Voltage-dependent anion-selective channel protein 1 |  |  |  |  |
| Q8K0C5 | Zymogen granule membrane protein 16 |  |  |  |  |
